# Tapping the potential of synthetic riboswitches: reviewing the versatility of the tetracycline aptamer

**DOI:** 10.1080/15476286.2023.2234732

**Published:** 2023-07-17

**Authors:** Daniel Kelvin, Beatrix Suess

**Affiliations:** a Fachbereich Biologie, TU Darmstadt, Darmstadt, Germany; b Centre for Synthetic Biology, TU Darmstadt, Darmstadt, Germany

**Keywords:** aptamer, riboswitch, synthetic biology, tetracycline, conditional gene expression

## Abstract

Synthetic riboswitches are a versatile class of regulatory elements that are becoming increasingly established in synthetic biology applications. They are characterized by their compact size and independence from auxiliary protein factors. While naturally occurring riboswitches were mostly discovered in bacteria, synthetic riboswitches have been designed for all domains of life. Published design strategies far exceed the number of riboswitches found in nature. A core element of any riboswitch is a binding domain, called an aptamer, which is characterized by high specificity and affinity for its ligand. Aptamers can be selected de novo, allowing the design of synthetic riboswitches against a broad spectrum of targets. The tetracycline aptamer has proven to be well suited for riboswitch engineering. Since its selection, it has been used in a variety of applications and is considered to be well established and characterized. Using the tetracycline aptamer as an example, we aim to discuss a large variety of design approaches for synthetic riboswitch engineering and their application. We aim to demonstrate the versatility of riboswitches in general and the high potential of synthetic RNA devices for creating new solutions in both the scientific and medical fields.

## Introduction

In recent decades, synthetic biology has become a major source of applications and solutions in the scientific, medical and industrial fields. As a result, a more complex set of challenges has emerged. These can range from the rapid and simple regulation of individual genes within complex metabolic pathways to the detection of specific small molecules with high affinity and specificity.

Riboswitches are genetic elements that can address these challenges. Naturally occurring riboswitches are highly structured regulatory RNA elements located in the 5’ UTR of several bacterial mRNAs that control a variety of different metabolic pathways [1]. They consist of an aptamer domain and an expression platform (e.g. incorporation of the ribosome binding site). The conformational changes induced by ligand binding to the aptamer domain affect the expression platform and can alter gene expression through different mechanisms, including transcriptional attenuation, sequestration of the ribosomal binding site, or ribozyme-mediated degradation [1–5]. Thus, it is possible to control gene expression at the RNA level without the need for additional regulatory factors (e.g. transcription factors).

The efficiency and simplicity of these naturally occurring principles inspired the development of synthetic riboswitches [6–8]. Unlike their natural counterparts, which are mostly found in prokaryotes, synthetic riboswitches have the added advantage of being easily adaptable to eukaryotic cell systems. These new designs not only utilized established riboswitch mechanisms, but also introduced new ways to regulate gene expression at both the transcriptional and translational levels [9,10]. Engineered riboswitches are designed to regulate gene expression as efficiently as possible and can be used strain independent in a wide variety of applications. Several promising projects have been published in recent years, and the major challenge now is to develop these designs into well-functioning devices that are practical for widespread application.

The aptamer domains can be those of natural riboswitches (currently limited to 55 classes) [11,12], but also de novo selected aptamers. Aptamers can be selected *in vitro* in a process called Systematic Evolution of Ligands by EXponential enrichment (SELEX) [13,14]. During this process, a large pool of partially randomized RNA sequences is brought into contact with an immobilized target molecule. RNAs that specifically recognize the target are eluted, amplified and re-incubated with the ligand. Non-binding RNAs are removed by washing steps. The process is iterative and typically takes several rounds. The resulting aptamers can bind to their ligands with up to picomolar affinities [15], some of which are comparable to monoclonal antibodies. In addition, aptamers can discriminate between chiral molecules and recognize a single epitope of their target molecule. Hydrogen bonds, electrostatic and van der Waals contacts, and the stacking of aromatic rings allow for highly affine ligand binding [16–18]. The ligands can be a wide variety of molecules [19]. Proteins, peptides, small molecules or other pharmaceutically active substances are conceivable. While most aptamers exist in a pre-structured state and do not change conformation upon ligand binding, some can undergo structural changes [20]. This ability has been shown to be important in the design of synthetic riboswitches.

The tetracycline aptamer has been used to design several synthetic regulatory devices in a variety of projects and was one of the first aptamers to be utilized for the design of a synthetic riboswitch. Using the tetracycline aptamer as an example, this review will focus on highlighting the potential of synthetic riboswitches as genetic regulatory devices in various biological systems and describe several new implementations of the aptamer.

## The tetracycline aptamer – selection and characterization

**Figure 1.**
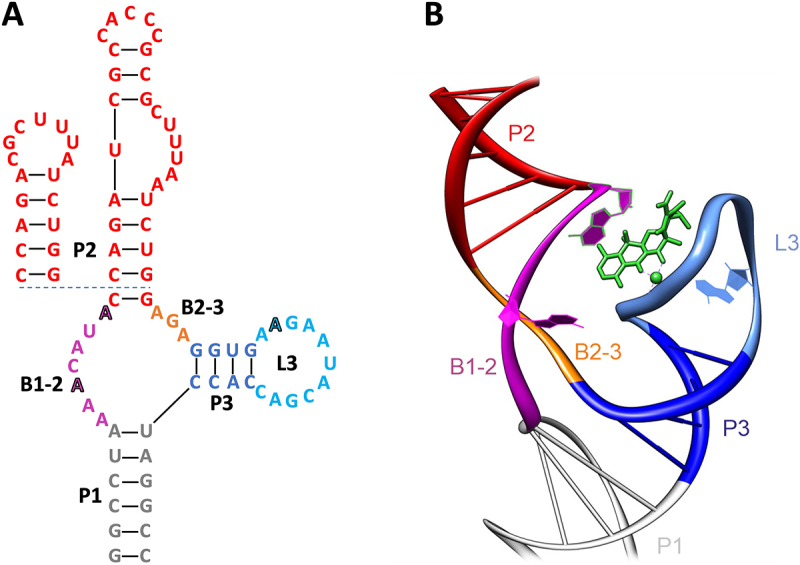
Depiction of the tetracycline aptamer cb32 in (A) 2D and (B) 3D. The scaffold of the aptamer is comprised of the three stems P1 (grey), P2 (red) and P3 (blue). The sequences of the basal stem P1 and the stem P2 can be fully exchanged (see minimer variant, inlet) and are only required as a scaffold for the structure. The stems are connected via the bulges B1–2 (purple) and B2–3 (orange), which together with the loop L3 (light blue) form the binding pocket for tetracycline (green). Nucleotides relevant for tetracycline binding have been highlighted.

The tetracycline aptamer was selected in 2001 using an RNA pool containing sequences of 74 random nucleotides flanked by constant regions [6] (113 nt in total). The target molecule tetracycline was coupled to an epoxy-activated sepharose column under alkaline conditions and brought into contact with the 5’-radiolabeled RNA. The column was then washed several times, with the number of column wash steps increasing every few rounds to increase stringency. Elution was initially performed with 100 µM tetracycline, which was reduced to 10 µM after round 8 to further increase stringency. A significant enrichment of sequences eluted specifically with tetracycline was observed after 15 rounds. 44 candidates from rounds 13 and 14 were cloned and sequenced. This led to the discovery of sequence cb28, which binds to tetracycline with high affinity and was used to generate a truncated 60 nt minimer version of the aptamer. Sequence cb32, which differs from cb28 by only one point mutation, was found to be the most biologically active candidate in terms of regulation [21] (Figure 1A).

Initial chemical and enzymatic probing of the tetracycline aptamer revealed a structure consisting of three helices P1-P3 connected in an inverted h-shape by two single stranded regions B1–2 and B2–3 (Figure 1). Stems P2 and P3 are closed by loops L2 and L3. All stems are pre-structured in the unbound state of the aptamer [9,15]. Nucleotides important for tetracycline binding were identified in B1–2, B2–3 and L3. The closing stem P1 as well as P2 are interchangeable and only relevant for maintaining the structural integrity (scaffold) of the aptamer. The crystal structure of the tetracycline aptamer was solved in 2008 and provided a detailed description of the tertiary structure of the aptamer and its binding properties [22] (Figure 1B). The two helices P1 and P3 are stacked. Ligand binding to nucleotides of B1–2 and B2–3 leads to the formation of an irregular helix on which P2 is based. When the aptamer is bound to the ligand, loop L3 interacts with the small ‘notch’ of the irregular helix. The nucleotides of B1–2 and B2–3 form non-Watson-Crick base pairs with three nucleotides of L3, resulting in the formation of a binding pocket in the form of a non-canonical pseudoknot in the presence of tetracycline.

The presence of magnesium ions is essential for this process, as tetracycline binds to the aptamer as a magnesium chelate. The magnesium ions are used to complement the formation of a pre-structured state of the aptamer and thus indirectly assist in the recognition of the ligand [23,24]. *In vitro* Thermodynamic characterization proved that the tetracycline aptamer binds to its ligand in an equimolar stoichiometry with a K_d_ as low as 770 pM [15]. A detailed characterization of the ligand binding kinetics suggests a ligand-induced stabilization of the closing stem P1 (B. Suess, personal communication). In addition, the aptamer was shown to be highly specific for tetracycline and to bind even its similar derivatives such as doxycycline, which differs from tetracycline only by the position of one hydroxyl group, with extremely low efficiency [6] (the binding efficiency is about 1000 times worse).

## The tetracycline riboswitch – conditional control of translation initiation in yeast

**Figure 2.**
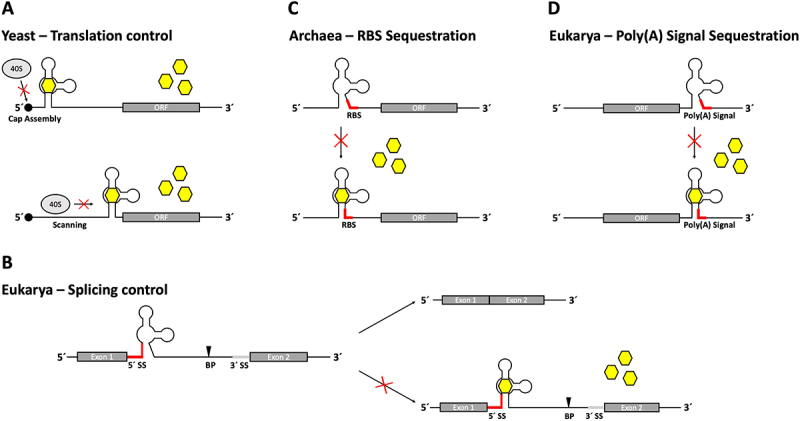
Tetracycline aptamer control mechanisms. (A) In yeast, positioning of the riboswitch construct within the 5’ UTR of the mRNA determines the type of translation regulation in the presence of tetracycline. Placement of the riboswitch near the 5’ cap structure hinders binding of the 40S ribosome subunit and thereby cap assembly of the ribosome, while codon-proximally placed constructs block ribosomal scanning. (B) Regulation of mRNA splicing is achieved by using the tetracycline aptamer to control accessibility of the 5’ splice site (SS). Incorporation of the 5’ SS sequence into the P1 stem of the riboswitch hinders recognition in the presence of tetracycline through stem formation. The branch point (BP) can therefore no longer initiate the first nucleophilic attack and the transcript is not spliced. (C) In archaea, sequestration of the ribosome binding site (RBS) through ligand-binding induced stem formation of the riboswitch prohibits assembly of the ribosome, thus inactivating translation. (D) Control over polyadenylation is exerted through incorporation of the AAUAAA hexamer polyadenylation signal (poly(A) signal) into the P1 stem of the tetracycline riboswitch. Positioning the riboswitch in the 3’ UTR of an mRNA enables sequestration of the poly(A) signal through ligand-binding induced stem formation, which impairs mRNA polyadenylation and results in faster degradation of the transcript.

One of the most established applications of the tetracycline aptamer is its function in controlling gene expression at the level of translation initiation in yeast [21]. Insertion into the 5’ UTR of an mRNA results in a dose-dependent decrease in gene expression in the presence of tetracycline (Figure 2A). The degree of regulation can be modulated by both the position of the aptamer within the 5’ UTR and its thermodynamic stability. The stabilization of the closing stem P1 (e.g. by additional base pairs) increases the regulation, but is accompanied by an overall decrease of the expression level even in the absence of tetracycline. Since the length and nucleotide sequence of P1 can be varied, the expression level of the unbound state and the overall switching factor of the construct can be modulated. In addition, the position of the aptamer within the 5’ UTR of an mRNA affects the efficiency of regulation. A position close to the start codon generally leads to a better regulation than a position close to the cap. However, it impairs the expression level significantly more than a cap proximal insertion [21,25].

The mechanism of translation inhibition is different depending on the position. A cap proximally inserted aptamer inhibits the formation of the 43S ribosome pre-initiation complex. Start codon proximal tetracycline aptamers block mRNA scanning by the small ribosomal subunit, preventing the formation of the fully active 80S ribosome (Figure 2A). While being less efficient *in vivo*, it has been shown that cap-proximal tetracycline aptamers can reduce gene expression up to 50-fold when analysed in a yeast cell-free system [25]. These differences between *in vivo* and *in vitro* measurements of the tetracycline aptamer’s switching capabilities could be attributed to the accessibility of the aptamer-containing mRNA within intact cells when covered by ribonucleoprotein particles (RNPs).

A rapid and simple method to improve the use of the aptamer system has been established by developing a tetracycline aptamer-containing regulatory cassette [26]. It contains a constitutive promoter (TDH3 or ADH1) to bypass endogenous expression of the gene of interest, followed by one or multiple copies of the tetracycline aptamer within a newly created 5’ UTR, an HA-tag for protein detection by Western blot and a *loxP*-flanked kanamycin resistance gene as a selection marker for chromosomal integration [26]. The device can be easily placed upstream of an endogenous target gene of interest through homologous recombination after amplification of the entire regulatory element by PCR using a set of gene-specific primers. It allows strain-independent control of gene expression as no regulatory proteins are required.

The regulatory cassette was used to control with five different essential yeast genes (*NEP1, NOP8, NOP14, PGI1* and *SEC1*). *NOP8* and *SEC1* were particularly interesting targets, as previous attempts to regulate these genes using conventional promoter-based systems had failed. All five genes were successfully expressed in the absence of tetracycline and could be down-regulated upon addition of the ligand, although *NEP1* and *PGI1* required the stronger TDH3 promoter to be expressed at sufficient levels. The insertion of more than one copy of the aptamer resulted in improved regulation. Three copies of the tetracycline aptamer were required to enable regulation of *NEP1*, demonstrating that an increased number of aptamer copies can enhance regulation [26]. The robust and efficient design of the tetracycline aptamer regulatory cassette have made it a widely used tool for the study of endogenous pathways [27–31].

As regulation was successfully increased by inserting more than one aptamer, a following approach attempted to engineer an optimized tetracycline-tandem riboswitch. Therefore, the closing stem P1 of the 3’ proximal aptamer was randomly mutated. It became clear that not only the length, but also the sequence composition was important for regulation. A machine learning approach was applied to find the best candidate. The method incorporated both convolutional neural networks and a random forest classification algorithm based on sequence and biophysical data. After four rounds of prediction and measurement, a construct with 40-fold inhibition of GFP expression in the presence of tetracycline was discovered [32].

## The tetracycline aptamer as a tool in splicing regulation

Splicing is considered to be the main source of human proteome diversity. This makes the low number of systems capable of conditionally controlling splicing even more surprising. The tetracycline aptamer was the first aptamer to be used for the control of pre-mRNA splicing. The aptamer was inserted close to the 5’ splice site of an actin intron located within a *gfp* reporter gene in yeast. Splicing was inhibited in the presence of tetracycline [33] (Figure 2B). Insertion near the branch point was also tested but was unsuccessful. Several constructs with varying proximity to the 5’ splice site were designed, but regulation was most successful when the entire 5’ splice site sequence was incorporated into the closing stem P1 of the aptamer. In contrast to translation regulation, the addition of the tetracycline aptamer did not lead to a decrease in gene expression in the absence of the ligand. This was attributed to the fact that although the aptamer is partially pre-formed in its unbound state, the P1 stem is not fully stabilized in the absence of tetracycline. This was demonstrated by structural probing. A cleavage signal from in-line probing within P1 indicated a breathing of the closing stem, which is abolished by tetracycline binding. It was proposed that this effect allows recognition of the 5’ splice site [33]. Regulation was also possible when the aptamer was inserted into the U3 intron under the same experimental conditions, demonstrating that this type of regulation is not specific for the actin intron. Insertion of multiple aptamer-controlled introns into a reporter gene resulted in even greater regulation, but required some distance in between the regulated introns.

Once the functionality of the tetracycline aptamer as a splicing regulator in yeast was established, new design strategies aimed to create more complex regulatory devices capable of controlling alternative splicing in mammalian cell lines. A cassette exon was designed that could be either retained or skipped by addition of tetracycline via the insertion of the aptamer near the 3’ splice site of the cassette exon [34]. The system was established in HEK293 cells using a synthetic three-exon minigene [35]. The 3’ splice site of the upstream intron was incorporated into the P1 stem of the aptamer, sequestering the splice site in the presence of tetracycline. This resulted in the skipping of the middle exon. The integration of this exon was designed to negate the presence of premature start codons that would lead to translation termination or nonsense-mediated mRNA decay. As a result, the coding sequence of the mRNA was correct only when this middle exon was skipped. It was demonstrated that the system was always successful in regulating exon skipping and acted in a dose-dependent manner in several different mammalian cell lines (HEK293, HeLa, MCF-7 and A-549).

The aptamer-controlled exon was then tested with several genes. The luciferase gene *luc+* was divided into two exons by insertion of the *bgl2* intron from the human ß-globin gene. Several versions of the exon controlled by aptamers of different stability were integrated into the middle of the *bgl2* intron. The *bgl2* intron with the aptamer-controlled exon was also inserted into the human *MAX* gene in the natural exon-exon-junction of exons 3 and 4. In both cases, expression of the correct protein was detected in the presence of tetracycline [34]. The aptamer-controlled exon was also used to engineer a kill switch by inserting it into the CD20 gene, which expresses a surface receptor that can induce cell death upon contact with the therapeutic antibody rituximab. After stable integration of the aptamer-controlled CD20 gene into HeLa cells, addition of rituximab resulted in cell death in the presence of tetracycline. This effect was not observed when the cells were treated with doxycycline, a chemically similar derivative that does not bind the aptamer, demonstrating the specificity of the system. The aptamer-controlled exon was later used to elucidate the function of CD20 as a membrane protein in B cells [36]. The CRISPR/Cas9 system was used to stably integrate the control device into the CD20 encoding *MS4A1* gene. This allowed conditional control of endogenous CD20 expression in the presence of tetracycline, revealing the role of CD20 as a key regulator of B cell surface marker proteins and the B cell resting state [36].

In another approach, an engineered *hluc+* luciferase gene was designed which contained the aptamer close to the 5’ splice site of an intron [37]. Placement of the 5’ splice site into the P1 stem of the tetracycline aptamer was tested on both the 5’ and the 3’ sides of the stem. Placement of the 5’ SS into the 3’ side of the stem resulted in more efficient regulators with less exon skipping in the absence of the ligand. This was attributed to less potential structural interference from the aptamer being in close proximity to the splice site, as it was now retained with the exon after splicing. It also resulted in the aptamer itself being able to exist without any additional exon sequence, since its position allowed it to become the exon itself. The stop codons contained within the aptamer sequence were placed in frame with the *hluc+* gene to prematurely stop translation in the absence of tetracycline. While this system was developed in HeLa cells, its functionality was also tested in the model organism *Caenorhabdidis elegans*.

## Synthetic riboswitches exerting control over the ribosome binding site

The tetracycline aptamer was established as a regulatory control element for gene expression in all domains of life, including archaea. Control of translation initiation was demonstrated in the methanogenic archaeon *Methanosarcina acetivorans* [38]. The tetracycline aptamer was inserted into the 5’ UTR of a *ß-lactamase* reporter gene, enabling it to control the accessibility of the ribosome binding site (RBS, Figure 2C). Following the information gained from splicing regulation, which implied that the positioning of a regulatory sequence within the closing stem of the aptamer could mediate regulation [33], the RBS was incorporated into the P1 stem. The accessibility of the included sequences (RBS, splice site) is the result of the P1 stem being less stable in the absence of tetracycline [20,33]. Ligand binding leads to stabilization of P1, which in turn masks the RBS. The overall stability of the construct and the positioning of the RBS within P1 influence regulation. In the end 11-fold inhibition of gene expression in the presence of tetracycline was achieved.

This principle of masking the RBS was also used in a different approach by positioning the RBS in a hairpin loop in between the tetracycline aptamer and a *gfp* reporter gene in *E. coli* [39]. Tetracycline binding results in destabilization of the hairpin, thereby unmasking the RBS and inducing gene expression. The resulting construct was established in a cell free system to serve as a biosensor for tetracycline.

## The tetracycline aptamer as a binding domain of more sophisticated riboswitches

Designing synthetic riboswitches is a challenging process and several different attempts have been made over the years to find suitable design strategies. So far, this review has presented design strategies using only the tetracycline aptamer as the switching element. However, there are also approaches where the aptamer has been used as a binding domain for more complex riboswitch designs. Both rational design and computational approaches have been utilized for such riboswitch design strategies.

A first rational design approach aimed to alter the ligand specificity of riboswitches by replacing the aptamer domain of a naturally occurring riboswitch with other aptamer domains. The goal was to use the expression platform of a riboswitch as a chassis for the creation of new chimera riboswitches that can bind to and regulate with different ligands [40,41]. Three different riboswitches controlling the genes *metE*, *yitJ* (ligand: S-adenosylmethionine, SAM) and *lysC* (ligand: lysine) in *Bacillus subtilis* were selected for their expression platforms. These candidates were chosen because their individual structures allow a clear distinction between the aptamer domain and the expression platform, thus enabling exchangeability of the aptamer domain without loss of function. All candidates modulate transcription termination via an anti-terminator/terminator principle that relies on ligand-binding induced stem stabilization, which incorporates parts of the anti-terminator sequence leading to the formation of the terminator structure. The P1 helix of the expression platform, which incorporates the anti-terminator sequence, directly connects to the aptamer domain, enabling ligand-binding dependent control over transcription termination. The aptamer domain of each riboswitch was replaced with several different naturally occurring aptamer domains, all of which yielded functional riboswitches with specificity for their new ligands. This was also repeated with the tetracycline aptamer to show that the modularity of this system is not dependent on natural aptamer domains and resulted in functional chimeras with specificity towards tetracycline. The performance of this new tetracycline riboswitch could be tuned by modulating the stability of the P1 helix.

Using a computational approach, an algorithm based on secondary structure prediction was used to find sequences that were partially complementary to the tetracycline aptamer and, when combined with it, would lead to the formation of a terminator hairpin loop in the absence of tetracycline. Addition of tetracycline induced the formation of the tetracycline aptamer, opening the terminator structure and allowing transcription [42]. These constructs were inserted into the 5’ UTR of the reporter gene *bgaB*. After an initial prediction of three different sequences, several modifications were made to the riboswitch sequence based on experimental data to increase its efficiency. In addition, it was shown that placing several of these constructs in a row (dimers, trimers) further increases the efficiency of regulation.

## Catalytically active – tetracycline responsive ribozymes

RNA as a regulatory device is not limited to gene regulation through structural changes and sequence sequestration. A special class of RNA sequences called ribozymes possess catalytic properties that allow them to undergo self-cleavage without any secondary factors [43]. Over the past few decades, several types of ribozymes have been discovered [44,45]. By fusing a catalytically active ribozyme domain (e.g. hammerhead, HDV, twister ribozyme) with a ligand-binding aptamer domain, a new type of regulatory device is created [46,47]. The catalytic activity of the device can then be controlled by binding of the ligand to the aptamer domain. These constructs are capable of functioning as riboswitches by enabling ligand-controlled self-cleavage of RNA sequences, thus providing a catalytic approach to gene regulation.

**Figure 3.**
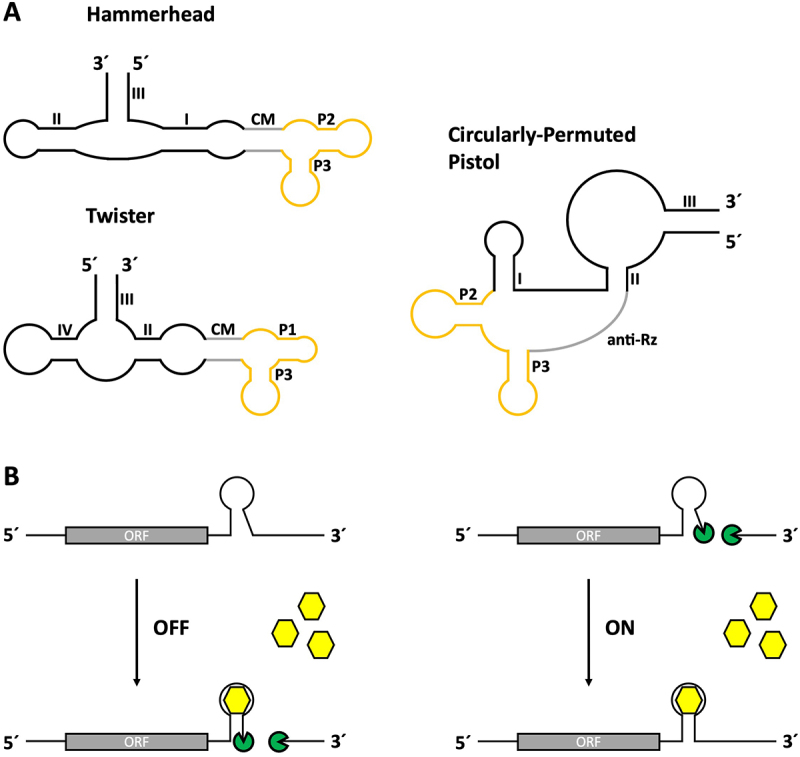
Tetracycline aptamer-controlled self-cleaving ribozymes. (A) The tetracycline aptamer has been combined with different ribozyme types to create functional aptazymes. A combination with the Hammerhead ribozyme N79 from *Schistosoma mansoni* was created by fusing the tetracycline aptamer at its P1 stem to stem I of the ribozyme, whose loop interactions with stem II are required for cleaving activity *in* *vivo*. This design was later improved by integrating three essential nucleotide positions of the original ribozyme sequence (71 U, 72A, 73C) into the P1 stem region of the tetracycline aptamer and randomizing the connecting region to find an optimized communication module (CM). Another design strategy utilized a circularly-permuted pistol ribozyme whose original stem III had been opened to create new 5’ and 3’ ends, whereas the former ends were connected via the sequence of the tetracycline aptamer. Instead of the P1 stem, an anti-ribozyme sequence (anti-Rz) was added to form a new stem with the ribozyme in the presence of tetracycline, thereby controlling the self-cleaving activity of the aptazyme. The third combination involved the Twister ribozyme, whose stem I was connected to stem P2 of the tetracycline aptamer. Stem I was chosen due to previous experiments with other aptamers showing promising results for the creation of functional aptazymes. Randomization of the connecting region and subsequent screening led to the discovery of several functional OFF-switch variants. (B) Regulation mechanism of self-cleaving aptazymes. Depending on the structure formation of the construct, binding of the ligand can either result in stabilization of the ribozyme, inducing cleaving activity (OFF-switch), or lead to destabilization, causing loss of cleaving activity (ON-switch). Cleaved mRNAs no longer possess a protective poly(A)-tail and are therefore subjected to degradation via enzymatic processes.

Among others, the tetracycline aptamer has been used in a wide variety of ribozyme designs. The first design strategy was to fuse the aptamer to the full-length hammerhead ribozyme N79 from *Schistosoma mansoni* via a randomized linker domain [48] (Figure 3A). The linker sequence was placed at stem I of the ribozyme because interactions of its loop region with the loop of stem II are required for cleavage *in vivo* [49]. The linker region was randomized and the resulting sequence pool was subjected to several rounds of *in vitro* selection to find constructs that could only cleave in the presence of tetracycline (Figure 3B). This led to the discovery of fast cleaving ribozymes capable of achieving dynamic ranges of up to 300-fold increased cleaving activity in the presence of tetracycline *in vitro*. Some constructs reached cleaving activities comparable to and even higher than those of the original hammerhead ribozyme N79, indicating that the addition of the tetracycline aptamer did not negatively affect the catalytic activity. The best performing aptamer-controlled ribozymes were then tested *in vivo* by insertion into the 3’ UTR of a *gfp* gene in *S. cerevisiae*. While the constructs were modestly functional in yeast, no regulatory capabilities were observed in mammalian cell lines. In another attempt, a theophylline and the tetracycline aptamer were coupled to stem I and II of the hammerhead ribozyme using different communication module designs [50] and later used to simulate different types of Boolean logic gates [51] utilizing *gfp* as a reporter gene in yeast.

The first tetracycline-responsive hammerhead ribozymes capable of regulating gene expression in HeLa cells were designed using a rational design approach [52]. An ON-switch design based on the disruption of loop-loop interactions of the hammerhead ribozyme was chosen. This included the integration of three essential nucleotide positions from the original ribozyme sequence into the P1 stem of the tetracycline aptamer fused to stem I of the ribozyme. The idea behind this rational design approach was that the aptamer would be flexible enough to keep these positions accessible to the ribozyme in the absence of tetracycline, thus allowing loop-loop-interactions and consequently cleavage (Figure 3B). Addition of the ligand then stabilizes the structure, resulting in sequestration of the required nucleotide positions. This would render the ribozyme inactive by disrupting the necessary loop-loop interaction. Insertion of aptamer-controlled ribozyme sequences with this design into the 3’ UTR of the *Renilla* luciferase gene *hRluc* and subsequent transfection of HeLa cells revealed ribozymes capable of inducing reporter gene expression in the presence of tetracycline. In dose-dependency measurements the most active candidate K19 reached up to 9-fold activation in the presence of tetracycline. HeLa cells were also used for stable genomic integration of an *egfp* reporter gene containing a tetracycline hammerhead ribozyme in its 5’ or 3’ UTR. The ribozymes proved to be more efficient when placed in the 3’ UTR. Ribozymes in the 5’ UTR interfered with gene expression even in the absence of tetracycline and resulted in a lower dynamic range.

The ribozyme K19 was later used to control the expression of mitochondrially targeted zinc finger-nucleases (mtZFNs) to combat mitochondrial diseases [53]. The premise was that cells containing both mutant and wild-type mitochondrial DNA (mtDNA) could be rescued by degrading the mutant mtDNA through site-specific DNA cleavage via mtZFNs, resulting in the enrichment of wild-type mtDNA. A K19 controlled mtZFN caused a strong shift towards wild-type mtDNA in the presence of tetracycline. More importantly, the control of mtZFN expression also helped to reduce the occurrence of side effects due to unwanted catalysis by the mtZFN at higher expression levels, thus improving the efficiency of the system.

A more complex application was demonstrated in the model organism *C. elegans* [54]. K19-driven reporter gene expression was found to be both rapid in its response time to the addition of tetracycline (upregulation of transcript levels after 12 h) and functional in all developmental stages of the nematode. Therefore, the system was used for the generation of inducible disease models. Both a functional Huntington’s disease mimicking worm strain containing a K19-driven mutant of huntingtin exon 1 with an extended polyglutamine stretch (*n* ≥ 35) and a neurologically defective unc-119(ed3) worm strain with a tetracycline-inducible Cbr-unc-119 rescue plasmid were established. The advantages of using the tetracycline ribozyme for these models lie in its innate compactness (only short sequence insertions and no other auxiliary factors required) and its ability to exert tight temporal (response time) and spatial (tissue-specific promoters) control over gene expression. These results can be considered a breakthrough as they establish K19 as a functional and broadly applicable ribozyme.

The ribozyme K19 was also used to control Adeno-associated viral (AAV) vector-mediated transgene expression in mice [55]. The goal was to improve AAV-mediated gene therapy by providing a greater degree of control over the expression of a therapeutic protein through transient dynamic induction of transgene expression in the animal. Riboswitches would be best suited for this role as they do not require the introduction of additional proteins that may cause immunogenic reactions in the patient. The secreted anti-Fluorescein isothiocyanate tandem single chain variable fragment anti-FITC scFv antibody and an intracellularly expressed nano-luciferase cNLuc were used as reporter genes. Administration of tetracycline induced the expression of both reporters in the tissues of treated mice, with anti-FITC scFv antibody levels reaching an up to 15-fold increase after several hours compared to the baseline expression without tetracycline. The comparatively lower increase in cNLuc expression (3.3-fold in liver tissue) was attributed to the accumulation of the protein in the cells prior to induction. As a result, the constantly secreted anti-FITC scFv antibodies, which have a shorter half-life than cNLuc, provide a more dynamic and accurate representation of riboswitch activity. Expression could be controlled in a dose-dependent manner by varying the amount of administered tetracycline.

When comparing several different aptamer-controlled ribozymes for the control of therapeutic transgenes, K19 was identified as the best candidate [56]. An optimized construct containing two ribozymes connected by a 100 bp linker was shown to be successful in regulating AAV-mediated expression of the transgene VEGF-B in human 293T cells.

Another approach aimed to improve the design principle of tetracycline ribozymes through optimization of the communication module sequence [57]. A scoring system was developed based on a dataset of tetracycline ribozymes with different communication module sequences in between the aptamer [22] and the hammerhead ribozyme N107 [58], focusing on three important factors: The number of hydrogen bonds of each base pair, their stacking interactions with neighbouring bases and their distance to the enzymatic core of the hammerhead ribozyme. These factors were determined by analyzing a variety of tetracycline ribozymes (OFF-switches) with manually generated communication module sequence variations. It was discovered that placing base pairs with fewer hydrogen bonds in close proximity to the ribozyme resulted in ribozymes with increased basal expression. All three factors were combined to assign a weighted hydrogen bond and stacking score (WHSS) to each communication module. Base pairs directly adjacent to the aptamer are also relevant, as the binding pocket requires a certain degree of stability to ensure a sufficient decrease in gene expression in the presence of the ligand. Several ribozyme variants were generated based on the new design principles. Fusing the tetracycline aptamer to the hammerhead ribozyme in a circularly permuted orientation by closing the tetracycline P1 stem with a stem loop and instead connecting the aptamer to the ribozyme via an open P2 stem was also tested. These circularly permuted ribozymes exhibited high dynamic ranges when following the previously established design principles for communication modules, with one construct outperforming all previously generated Tc-P1 ribozymes (Tc40). This variant was used to exert conditional control over retroviral transgene expression using an adeno-associated virus vector expressing either Gluc luciferase or proteins relevant for the gene therapy of haemophilia B and rheumatoid arthritis (human coagulation factor IX and etanercept). Tight control was achieved in both transduced HeLa cells and BALB/c mice (Gluc only), whose transduced gastrocnemius muscle cells exhibited a dynamic range of fluorescence of up to 6.9-fold from before to after treatment with tetracycline *in vivo*.

The hammerhead ribozyme is not the only scaffold used to generate tetracycline-responsive ribozymes in mammalian cell lines. A circularly permutated variant of the pistol ribozyme [59] has been used as an alternative ribozyme scaffold [60]. A tandem aptamer-ribozyme architecture was designed in which the aptamer, containing portions of the ribozyme sequence and an anti-ribozyme sequence in its basal stem, was placed either upstream of the ribozyme or in the newly created flexible linker region (Figure 3A). Binding of the ligand to the aptamer results in structural stabilization and formation of the ribozyme-complementary stem, thereby excluding the structure of the functional ribozyme and preventing self-cleavage.

The tetracycline aptamer was also attached to the twister ribozyme [61] (Figure 3A). An interesting high-throughput approach was utilized for this design. A library of semi-randomized aptamer-controlled ribozyme sequences containing a randomized region in between the tetracycline aptamer and the twister ribozyme was inserted into the 3’ UTR of a reporter gene and expressed in HEK293 cells in the absence and presence of tetracycline. The RNA was extracted, reverse transcribed into cDNA and the ribozyme sequences were amplified by PCR and sequenced. Comparison of cDNA levels allowed an assessment of their switching capabilities.

## Novel riboswitch mechanisms for mammalian synthetic biology

**Figure 4.**
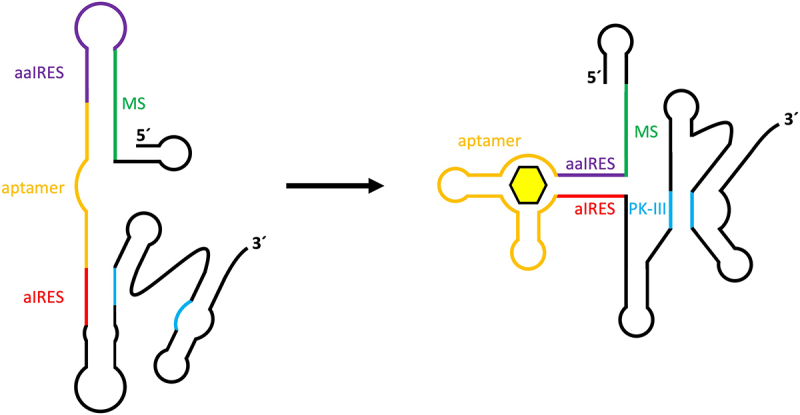
Construct design for tetracycline aptamer-controlled IRES-mediated translation regulation. An internal ribosome entry site (IRES) contains several sequences and structures that are required for enabling internal ribosome entry and translation. The formation of an important pseudoknot structure (PK-III) is hindered through complementary binding of an anti-IRES sequence (aIRES) in the absence of a ligand for the connected tetracycline aptamer. An anti-anti-IRES sequence (aaIRES), which is designed to disturb aIRES inhibition of PK-III formation, is itself inhibited by a modulator sequence (MS). Binding of tetracycline to the aptamer promotes stem formation between the aIRES and aaIRES sequences, thus enabling PK-III formation and subsequent activation of IRES-mediated translation.

The design of synthetic riboswitches capable of controlling translation is more complicated in eukaryotes than in prokaryotes due to differences in translation initiation. Whereas in prokaryotes the sequestration of the ribosome binding site provides an easy way to regulate gene expression, there are no such sequence motifs in eukaryotes, which rely on 5’ cap-mediated ribosome assembly. It has also been shown that attempts to control translation by inserting an aptamer into the 5’ UTR, as described for yeast, have only been successful in a few cases [62]. In an alternative approach, translation initiation in eukaryotes was efficiently controlled via the introduction of an internal ribosome assembly site (IRES) derived from an intergenic region of the *Plautia stali* intestine virus [63]. Assembly of the ribosome at the IRES enables the use of sequestration mechanisms for translation control similar to those found in prokaryotic systems. In this approach, 5’ cap-mediated translation is inhibited by the addition of a 5’ stem loop structure. A riboswitch was designed by adding a sequence complementary to important parts of the IRES (anti-IRES) for sequence sequestration, a sequence complementary to the anti-IRES (anti-anti-IRES) to re-enable IRES functionality, a modulating sequence to mediate between the two states and the aptamer itself to allow ligand-dependent control of switching (Figure 4). The entire construct was introduced into the 5’ UTR of a firefly luciferase gene to test functionality *in vitro* in wheat germ extract. While initial tests were performed with the theophylline aptamer, modification of the construct to instead incorporate the tetracycline aptamer, by calculating and adjusting the required free energy of the modulating sequence, resulted in a tetracycline-dependent IRES switch with the highest *in vitro* switching factor of all presented constructs.

The aptamer was also used to conditionally control polyadenylation in HeLa cells [64]. This work focused primarily on modifying the guanine-binding aptamer domain of the *B. subtilis xpt* riboswitch to sequester a polyadenylation signal (AAUAAA hexamer) located in the 3’ UTR of the *hRluc* luciferase gene in the presence of guanine. This was achieved by incorporating both the signal sequence and a complementary sequence into the basal stem of the aptamer. The system was also adapted to use the tetracycline aptamer, which proved successful in regulating polyadenylation via signal sequestration after additional stabilization of its P1 stem (Figure 2D). The tetracycline polyadenylation riboswitch was further used to control alternative polyadenylation by inserting two different poly(A) signals into the 3’ UTR of the *hRluc* luciferase gene and placing the proximal signal under its control. A more complex regulatory system was also created by positioning the aforementioned tetracycline ribozyme K19 upstream of the guanine polyadenylation riboswitch in the 3’ UTR, resulting in a Boolean logic gate [64].

In an unconventional approach the poly(A) signal was placed into the 5’ UTR of a luciferase reporter gene under the control of the tetracycline aptamer [65]. Gene expression is inhibited by cleavage of the poly(A) signal in the absence of tetracycline. While the first design approach relied on inserting the poly(A) signal into the sequence of a single aptamer, placement into the middle of a Y-shaped fusion of three aptamers resulted in even greater induction of gene expression in the presence of tetracycline. A construct combining this design with alternative splicing influenced by the stability of the aptamer structure yielded over 700-fold induction of gene expression. Experiments in human cell lines and mice proved the high efficiency and adaptability of the construct.

Another design focused on the conditional control of microRNA-mediated RNA silencing [66]. It consists of a microRNA target sequence (miR-T) and a complementary competing strand separated by the tetracycline aptamer. MicroRNA binding to miR-T induces RNA silencing via the RNA-induced silencing complex (RISC), while ligand binding to the aptamer induces stem formation between miR-T and the competing strand, thereby masking miR-T and preventing microRNA binding. The entire construct was inserted into the 3’ UTR of a luciferase reporter gene and two different designs with miR-T on either the 3’ or 5’ side of the aptamer were tested. In the end, a combined dual-input switch containing an aptamer and half of the competing strand both 3’ and 5’ of miR-T proved to be the most efficient design.

## The tetracycline aptamer *in trans*

All approaches presented so far have focused on regulatory concepts where the aptamer is placed into the mRNA of the gene of interest (*in cis*). However, aptamers can also affect gene expression *in trans*. *In trans* acting devices can control prominent mechanisms such as RNA interference or CRISPR/Cas. Both focus on protection from viral influence by identifying and terminating foreign nucleic acids using short RNA fragments (micro (mi)RNA, small interfering (si)RNA and (tra)crRNA) as identification markers. Both methods have become important tools for targeting gene expression by introducing synthetic sequences (e.g. short hairpin (sh)RNA or single guide (sg)RNA). The availability of such small regulatory RNAs can be controlled by aptamers in a ligand-dependent manner.

One approach attempted to regulate gene expression by aptamer-mediated ligand-inducible RNA interference [67]. An aptamer sequence was inserted into an shRNA, the synthetic precursor of siRNA. The terminal loop was replaced with the aptamer and an additional strand competing with the shRNA stem was added. The construct was designed as an ON-switch with two distinct conformations. In the active state, the shRNA stem forms, resulting in siRNA production and gene silencing. Ligand binding to the aptamer induces base pairing with the competing strand, disrupting the shRNA and inactivating RNA interference. The tetracycline aptamer was used during modelling and tuning to evaluate the effect of aptamer size on minimal basal expression in the active conformation.

In addition to RNA interference, the CRISPR/Cas system offers another interesting approach to control gene expression *in trans*. An sgRNA was equipped with a tetracycline aptamer sequence at its 3’ end to create a ligand-responsive CRISPR/Cas regulatory device [68]. The sequence of the aptamer stem was designed to be complementary to the guide region of the sgRNA, resulting in sequence sequestration in the absence of tetracycline. Ligand binding then leads to refolding of the sgRNA, releasing the guide sequence and allowing the construct to bind directly to the target gene. A catalytically inactive version of the Cas9 endonuclease (dCas9) was used to silence transcription. Alternatively, the system could activate transcription by using a fusion protein containing an additional transcription factor (dCas9-VP64). Experiments in HEK293T cells showed tetracycline-responsive increases and decreases in gene expression of the target protein depending on the type of dCas9-based regulator. This design approach, originally based on the tetracycline aptamer, was then used to create various types of logic gates and intracellular networks using aptamers that respond to intracellular proteins and signals.

Other rationally designed trans-acting regulatory devices use the tetracycline aptamer for application in yeast [69]. These so-called antiswitches consist out of a ligand-sensing aptamer domain, an antisense domain complementary to the mRNA of the gene of interest and a connecting stem region. This linker is designed to sequester the antisense domain in the absence of the ligand. Control over gene expression is exerted through conformational changes induced by ligand binding to the aptamer, resulting in the antisense domain being favoured in a single-stranded state. Regulation is achieved through translational inhibition by binding of the antisense domain to a site on the targeted mRNA containing the start codon of the gene of interest.

## Conclusion and future challenges

Conditional control of gene expression is well established and widely used. However, it is mostly limited to transcriptional control and requires the expression of auxiliary factors that sometimes cause metabolic burden or unwanted immune responses. In addition, the genomic location sometimes limits the applicability. Therefore, alternative strategies that do not rely on transcription, regulatory proteins or genomic integration are needed for some applications. Synthetic riboswitches as RNA-based genetic control elements circumvent many of these shortcomings and thus represent an attractive alternative. They consist entirely of RNA, can recognize their ligand by themselves in an intrinsic binding pocket and do not require a protein for this purpose. Thus, they are sensor and regulator in one molecule, which makes them small in size and without significant metabolic burden. Moreover, they have been designed not only for bacteria, the natural source of such riboregulation, but for all three domains of life, and the design strategies now encompass an incredible diversity. Synthetic riboswitches have been developed not only to regulate transcription termination or translation initiation – the main mechanisms of the natural riboswitches – but also to control splicing, polyadenylation, mRNA stability or the accessibility of small regulatory RNAs – systems for which almost no conditional gene regulation systems have yet been described. Thus, in recent years there have been an enormous number of very interesting developments in the field.

Using the example of the tetracycline aptamer as a regulatory and sensory tool, the wide range of design strategies for riboswitch regulation was presented. Due to its small size, high ligand specificity, reliable functionality and easily modifiable sequence, the aptamer has attracted great interest and not only demonstrates its versatility, but also broadens the biotechnological toolbox, allowing the use of these devices in more complex applications in the future. Understanding the principles of the tetracycline aptamer has proven to be a key factor in its use in many different applications, and we have elucidated its structure and sequence to the point where we understand its composition and switching behaviour, as well as the underlying mechanisms involved in ligand binding.

However, the findings summarized in this review also point to the limitations of the field to date. In many applications, it has been empirically determined where the optimal insertion positions are, but context dependency and transferability are still a problem in some applications. Furthermore, the number of aptamer domains currently used for the design of synthetic riboswitches is very limited, as it has been shown that not only very good binding but also conformational switching is necessary for optimal regulation, and identifying new aptamer domains suitable for riboswitch engineering is far from trivial (reviewed elsewhere) [20]. However, examples such as the tetracycline aptamer insertion cassette engineered for yeast, which has been successfully applied by many groups, or the versatile application of the ribozyme K19 show that designs utilizing regulation by synthetic riboswitches can no longer be considered only as proof-of-concept studies, but have partially reached a stage of robust application.

The rapidly growing importance of synthetic RNA biology in both the scientific and medical communities requires fast and efficient approaches to the design of new genetic components. We have shown that a well-understood construct such as the tetracycline aptamer can be used in an extremely wide range of applications and adapted for uses not even imagined at the time of its creation. Continuing to push these ideas allows us to move beyond the proof-of-concept stage, as we have shown with the tetracycline aptamer and its use in endogenous gene analysis and control of key cellular factors. By creating more complex systems capable of performing their tasks with greater efficiency, we are enabling their use in new and compelling biotechnological and medical applications.

## Data Availability

Data sharing is not applicable to this article as no new data were created or analysed in this study.
